# Treatment of idiopathic pulmonary hemosiderosis in children: a systematic review

**DOI:** 10.3389/fped.2026.1982437

**Published:** 2026-10-08

**Authors:** PengCheng Liu, ZhiChao Lei

**Affiliations:** 1 Department of Pediatric Respiratory Medicine, Guiyang Maternal and Child Health Care Hospital, Guiyang, Guizhou, China; 2 Department of Critical Care Medicine, Guizhou Third People’s Hospital, Guiyang, Guizhou, China

**Keywords:** children, corticosteroids, diffuse alveolar hemorrhage, idiopathic pulmonary hemosiderosis, rituximab, treatment

## Abstract

**Background:**

Idiopathic pulmonary hemosiderosis (IPH) is a rare disease characterized by recurrent diffuse alveolar hemorrhage in children. Corticosteroids are the conventional first-line treatment, and immunosuppressive agents also constitute a mainstay of management. However, evidence is derived almost entirely from small observational studies, and no standardized recommendations exist.

**Methods:**

We systematically searched PubMed, Embase, Web of Science, the Cochrane Central Register of Controlled Trials, and Chinese databases (CNKI and Wanfang) from inception to 24 September 2026, with no language restrictions. Studies reporting treatment for IPH in children (0–18 years) were included; a narrative synthesis was performed.

**Results:**

A total of 149 studies were included. Corticosteroids were the first-line treatment, with 78.7% of the 494 children with IPH or undetermined etiology in the largest multicenter cohort (*n* = 567) receiving corticosteroid monotherapy. The overall relapse rate ranged from approximately 35% to 60% across small single-center cohorts, most commonly triggered by respiratory infections and corticosteroid tapering. Immunosuppressive agents, including azathioprine, hydroxychloroquine, cyclophosphamide, leflunomide, and 6-mercaptopurine, were added for refractory, steroid-dependent, or relapsing disease. Biological agents such as rituximab also emerged as promising steroid-sparing options. Adjunctive therapies included intravenous immunoglobulin, ECMO, arterial embolization, and iron chelation. In a single-center IPH cohort of 41 children, mortality was 27.3% (6 of 22 children followed).

**Conclusions:**

Corticosteroids remain the cornerstone of pediatric IPH treatment, with immunosuppressive or biologic agents reserved for refractory disease. Given the absence of high-quality evidence, multicenter prospective studies with standardized protocols are urgently needed.

**Systematic Review Registration:**

https://www.crd.york.ac.uk/prospero/display_record.php?ID=CRD420261486860, PROSPERO CRD420261486860.

## 1 Introduction

Idiopathic pulmonary hemosiderosis (IPH) is a rare disorder of unknown etiology (Orphanet: ORPHA:99931) characterized by recurrent diffuse alveolar hemorrhage (DAH), with hemosiderin deposition in the lungs and iron-deficiency anemia (1, 2). Although its pathogenesis remains incompletely understood, IPH is widely regarded as an autoimmune disease with a possible genetic predisposition, and respiratory tract infections typically act as triggers of recurrent hemorrhage rather than as the underlying cause (1, 2). Its estimated incidence is 0.24–1.23 per million; as the idiopathic subtype of DAH, IPH accounts for approximately 13% of DAH in children, with about 80% of cases occurring before 10 years of age (3).

The classical triad comprises hemoptysis, iron-deficiency anemia, and diffuse pulmonary infiltrates; however, the presentation is frequently atypical, and misdiagnosis rates as high as 73.8% have been reported in a large pediatric cohort (4). Recurrent alveolar hemorrhage may lead to progressive pulmonary fibrosis and respiratory failure, emphasizing the need for early diagnosis and timely treatment (1, 2). The diagnosis of IPH requires confirmation of diffuse alveolar hemorrhage, most often by bronchoalveolar lavage demonstrating hemosiderin-laden macrophages or by lung biopsy, together with exclusion of known secondary causes such as antineutrophil cytoplasmic antibody (ANCA)-associated vasculitis, systemic lupus erythematosus, anti-glomerular basement membrane disease, and celiac disease-related Lane–Hamilton syndrome (1, 2, 11, 29).

Pharmacotherapy is currently the cornerstone of IPH management, primarily comprising corticosteroids and immunosuppressive agents, which provide rapid symptom control during acute episodes and maintain remission during the chronic phase. Corticosteroid monotherapy is the mainstream initial therapy for most children (5). For children with corticosteroid-refractory disease, steroid dependence, or recurrent relapses, immunosuppressive agents such as cyclophosphamide, azathioprine, 6-mercaptopurine, hydroxychloroquine, and rituximab are commonly added (6). Nevertheless, relapse remains a major challenge in the treatment of IPH (7).

Despite the availability of these therapeutic options, there is currently no international guideline specifically addressing the management of pediatric IPH, and treatment evidence is derived almost exclusively from case reports, case series, and small retrospective cohorts, as no randomized controlled trials have been conducted for this rare disease. Moreover, a global survey of physicians found marked variability in the management of IPH, reflecting the absence of standardized protocols (8). Although several narrative reviews have summarized current knowledge of IPH (1, 2), and one systematic review has compared adult patients with IPH and Lane–Hamilton syndrome (9), these syntheses differ from the present review in three key respects. First, their study populations were either mixed or exclusively adult, whereas the present review focuses specifically on children and adolescents (0–18 years), in whom dosing, treatment response, and disease course differ from those in adults. Second, prior syntheses drew largely on English-language sources, whereas the present review searched six databases without language restriction and additionally included 19 Chinese-language studies. Third, no previous review has quantified pediatric treatment outcomes across a large body of studies; the present review synthesizes 149 studies and is the first to systematically quantify relapse and mortality outcomes in children. To the best of our knowledge, the present review is the first systematic review to synthesize treatment outcomes specifically in children with IPH, filling a critical gap in the current evidence.

Accordingly, this review addressed the following structured question: in children and adolescents aged 0–18 years with idiopathic pulmonary hemosiderosis, what treatment strategies have been reported, and what are their associated outcomes in terms of remission, relapse, mortality, and safety? Given that most included studies were single-arm and uncontrolled, findings were synthesized descriptively to characterize the available treatment options and their outcomes, rather than to compare the efficacy of one regimen against another. To answer this question, we systematically identified and synthesized the available evidence on the treatment of pediatric IPH to inform clinical decision-making and guide future research.

## 2 Materials and methods

### 2.1 Protocol and registration

This systematic review was conducted in accordance with the Preferred Reporting Items for Systematic Reviews and Meta-Analyses (PRISMA) 2020 statement (10). The review protocol was registered with PROSPERO (CRD420261486860) under the title “Treatment of idiopathic pulmonary hemosiderosis in children: a systematic review”.

### 2.2 Search strategy

We systematically searched PubMed, Embase, Web of Science, the Cochrane Central Register of Controlled Trials, and ClinicalTrials.gov from database inception to 24 September 2026, with no language restrictions. The complete PubMed search strategy is provided in the Supplementary Material. In addition, we searched the Chinese databases CNKI and Wanfang, and manually screened the reference lists of included studies and relevant reviews to identify additional records. We also searched the WHO International Clinical Trials Registry Platform (ICTRP) for ongoing trials, and identified one registered trial (NCT05937191) investigating leflunomide in pediatric IPH (Phase 1/2, *n* = 34, estimated completion December 2026).

### 2.3 Eligibility criteria

Studies were included if they: (1) enrolled children and adolescents aged 0–18 years with a diagnosis of idiopathic pulmonary hemosiderosis (IPH), including cases described as idiopathic diffuse alveolar hemorrhage (DAH) (these terms are conceptually synonymous); (2) reported treatment with corticosteroids alone or combined with immunosuppressive or immunomodulatory agents (azathioprine, hydroxychloroquine, cyclophosphamide, mycophenolate mofetil, 6-mercaptopurine, leflunomide, rituximab, intravenous immunoglobulin); and (3) reported at least one outcome of interest (remission, relapse, mortality, pulmonary function, or adverse events). We excluded studies of adults, secondary DAH (e.g., vasculitis, systemic lupus erythematosus, post-hematopoietic stem cell transplantation, Goodpasture syndrome, and other defined causes), and narrative reviews without original patient data. One amendment to the registered protocol warrants explanation. Although the PROSPERO protocol initially excluded single case reports, these were ultimately included because, for this rare disease, no randomized controlled trials exist and case reports and small case series constitute the vast majority of the available evidence; excluding them would have left the review unrepresentatively small and clinically incomplete. This decision lowered the overall level of evidence but improved the comprehensiveness and real-world representativeness of the review; the resulting low level of evidence is acknowledged in the limitations of the Discussion.

### 2.4 Study selection and data extraction

Two reviewers independently screened titles and abstracts, then reviewed the full texts of potentially eligible studies; disagreements were resolved by discussion or a third reviewer. The selection process is summarized in a PRISMA 2020 flow diagram. Data were independently extracted using a standardized form, including study characteristics, patient demographics, treatment regimen (agent, dose, route, duration), and outcomes. Outcome definitions were extracted from or adapted from the included studies. For the purposes of this review, relapse was defined as exacerbation of symptoms or worsening of imaging findings after remission (7); steroid dependence was defined as relapse upon corticosteroid tapering or discontinuation (4, 22); refractory disease was defined as persistent disease activity despite adequate corticosteroid therapy (1, 14); and remission was defined as resolution of symptoms with radiographic improvement (4, 7). Because the primary studies applied heterogeneous outcome definitions, these working definitions guided the narrative synthesis, and discrepancies are acknowledged where relevant.

### 2.5 Quality assessment

Methodological quality was assessed using the National Institutes of Health (NIH) Quality Assessment Tool for Case Series Studies; cohort studies were assessed with the Newcastle-Ottawa Scale (NOS), and any randomized trials with the Cochrane Risk of Bias 2.0 tool.

### 2.6 Data synthesis

Given the absence of randomized controlled trials and the expected clinical heterogeneity, a narrative synthesis was planned. Where three or more studies reported the same outcome, a single-arm meta-analysis of proportions was considered using a random-effects model with the Freeman-Tukey double arcsine transformation; heterogeneity was quantified using the I^2^ statistic.

### 2.7 Ethics approval

This systematic review was based entirely on previously published, de-identified data and did not involve direct contact with human participants; therefore, no ethics approval was required.

## 3 Results

### 3.1 Study selection

The systematic search identified 326 records from PubMed, 733 records from Embase, and 163 records from Web of Science, plus 6 records from Cochrane CENTRAL, 1 trial registration from ClinicalTrials.gov, and 20 records from the Chinese databases CNKI and Wanfang. An additional 7 records were identified through citation searching of reference lists. After removing 325 duplicate records, 931 records were screened by title and abstract, of which 782 were excluded. The remaining 149 records were assessed for full-text eligibility, and all 149 studies were included in the qualitative synthesis. The study selection process is shown in the PRISMA flow diagram (Figure 1).

**Figure 1:**
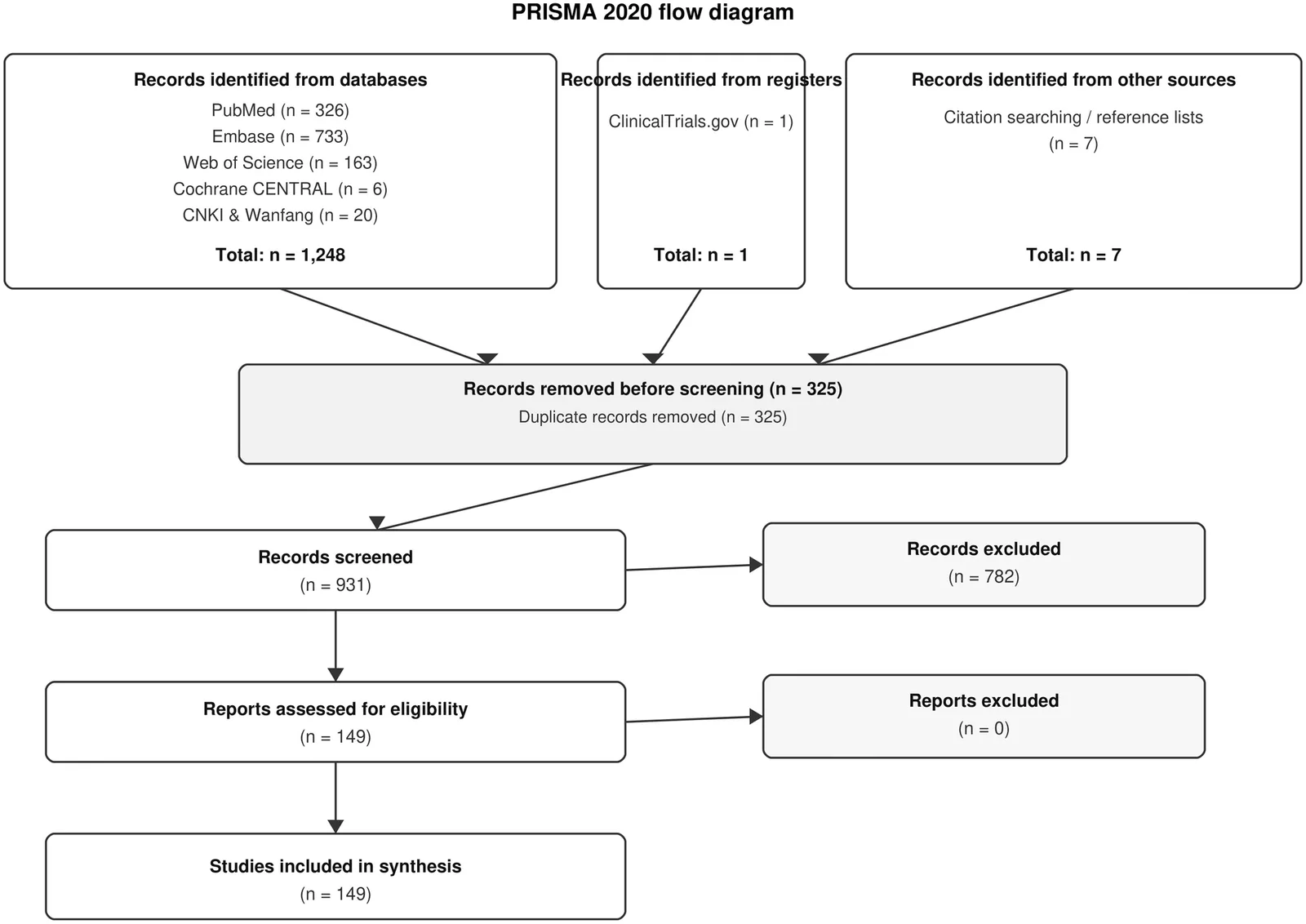
PRISMA 2020 flow diagram of study selection.

### 3.2 Study characteristics

The included studies spanned from 1959 to 2026 and originated predominantly from China, Europe, India, Japan, and South America. The vast majority were case reports and small case series, together with several retrospective cohort studies (11); no randomized controlled trials were identified. Sample sizes ranged from single cases to several hundred patients in the largest multicenter cohorts. The characteristics of representative included studies are summarized in Table 1, including additional cohort studies (34, 36). Methodological quality was formally assessed using the NIH Quality Assessment Tool for Case Series Studies and the Newcastle-Ottawa Scale for cohort studies. Because most included studies were single-arm case reports and case series without control groups, retrospective in design, and limited by small sample sizes, the overall quality of evidence was low and the risk of bias was high across the included studies; no study was rated at low risk of bias. Owing to the marked clinical and methodological heterogeneity (variable regimens, doses, and outcome definitions), a formal meta-analysis was not undertaken, and findings were summarized descriptively. Treatment of pediatric IPH has evolved over time: corticosteroids alone were the mainstay in the early decades; conventional immunosuppressants (azathioprine, hydroxychloroquine, cyclophosphamide, 6-mercaptopurine) were progressively introduced from the 1990s–2000s as steroid-sparing agents; and biological agents such as rituximab and newer agents such as leflunomide have emerged since the 2010s for refractory or steroid-dependent disease.

**Table 1:** Characteristics of representative included studies.

First author, year	Country	Design	*N*	Treatment (dose)	Key outcomes
Multicenter Collaborative Group, 2026	China	Multicenter cohort	567	CS alone 78.7% (among 494 with IPH/undetermined etiology); CS + IS 9.1%	Mortality 8.7% (DAH cohort); CS ≥3 mo lower mortality
Zang, 1995 (28)	China (Beijing)	Case series	245	CS; deferoxamine for ≥4 recurrences	Hemoptysis resolved in 1–2 wk
Zhang Y, 2019 (4)	China (West China)	Retrospective cohort	107	CS (prednisone 1–2 mg/kg/d)	Misdiagnosis 73.8%; 102/105 survived
Qiu, 2013 (31)	China (Zhejiang)	Cohort	41	CS	Mortality 27.3% (6/22); high bilirubin → poor prognosis
Zhang H, 2017 (15)	China (Xijing)	Cohort	36	Prednisone 1–2 mg/kg/d	Duration >3 yr did not reduce relapse
Yin, 2011 (13)	China (Qinghai)	Case series	35	MP 20–30 mg/kg/d × 3 d + IVIG 400 mg/kg/d × 5 d	All survived
Wang L, 2024 (7)	China (West China 2nd)	Cohort	32	CS; pulse CS	Relapse 59.4%; allergy HR 4.255
Chryssanthopoulos, 1983 (34)	Greece	Cohort	30	CS	Early prognostic criteria
Kabra, 2007 (35)	India (AIIMS)	Cohort	26	CS + HCQ	17/26 relapse-free; 4 added AZA
Taytard, 2013 (36)	France (RespiRare)	Cohort	25	CS; 13 added IS	23/25 satisfactory; 1 fibrosis; 1 death
Zhuo, 2020 (16)	China (Hunan)	Case series	19	CS; +AZA 5/7; +HCQ 3/3	AZA effective but AE; HCQ good alternative
Wang H, 2023 (6)	China (Sun Yat-sen)	Case series	46	Leflunomide+CS	Hb increased; steroid dose reduced
Terheggen-Lagro, 2022 (21)	Netherlands	Case series	2	Rituximab	Steroid-sparing; well tolerated >4 yr
Xu, 2017 (30)	China (Sun Yat-sen)	Case series	10	CS + leflunomide+MSC transplant	Complete remission in all; median FU 23 mo

Doses for intravenous cyclophosphamide and rituximab were largely extrapolated from adult or vasculitis regimens (14); azathioprine and hydroxychloroquine doses reflect pediatric usage. CS, corticosteroids; IS, immunosuppressants; MP, methylprednisolone; IVIG, intravenous immunoglobulin; AZA, azathioprine; HCQ, hydroxychloroquine; MSC, mesenchymal stem cells.

### 3.3 Corticosteroids

Corticosteroids were the first-line treatment across nearly all included studies (4). In the largest multicenter cohort of 567 Chinese children, among the 494 with IPH or undetermined etiology, 78.7% received corticosteroids alone as initial therapy (5). Oral prednisone at 1–2 mg/kg/day was the most commonly used regimen (4); high-dose corticosteroid therapy (≥1 mg/kg/day prednisolone equivalent) was associated with better outcomes, including a lower risk of ICU admission and a higher hemoglobin recovery rate, in one cohort (12). Acute severe hemorrhage was managed with intravenous methylprednisolone pulse therapy (20–30 mg/kg/day for 3 days), and a combination of methylprednisolone with high-dose intravenous immunoglobulin (400 mg/kg/day for 5 days) rapidly controlled pulmonary bleeding in a series of 35 patients, all of whom survived (13). Once clinical stability was achieved after 4–8 weeks, corticosteroids were tapered by 2.5 mg weekly (or 5 mg every other week) to a maintenance dose of 10–15 mg/day (adult dosing), and could be discontinued after 12–18 months of asymptomatic remission (14). Alternatively, one series recommended maintaining a low-dose prednisone of 0.5 mg/kg/day for at least 2 years (13). Liposteroid (dexamethasone palmitate incorporated in liposomes) has also been used, administered intravenously at 0.05–0.08 mg/kg/day for three consecutive days during exacerbations, followed by gradual tapering with lengthening intervals from weekly to biweekly to monthly, with favorable long-term outcomes (14, 24, 25). However, corticosteroids did not fully resolve the relapse problem: the duration of prednisone treatment did not influence the relapse rate (a study of 36 children reported no significant difference in relapse among patients treated for ≤1 year, 1–2 years, 2–3 years, or >3 years; *P* > 0.05) (15), and high-dose pulse corticosteroid therapy did not reduce the recurrence rate in a cohort of 32 children, whose overall relapse rate was 59.4% (7).

### 3.4 Conventional immunosuppressants

For children who respond inadequately to corticosteroids, are steroid-dependent, or experience recurrent relapses, conventional immunosuppressive agents are commonly added. Azathioprine is among the most frequently used: in a Chinese series of 19 children (16), it was combined with corticosteroids in 7 patients and achieved sustained remission in 5, although 3 patients discontinued it because of adverse effects (granulocytopenia in 2, gastrointestinal intolerance in 1) and were switched to hydroxychloroquine, which reduced relapse frequency in all 3. Azathioprine was used at 1.2–2.5 mg/kg/day (17), with baseline thiopurine S-methyltransferase (TPMT) testing recommended, as low TPMT activity is associated with an increased risk of bone marrow toxicity (14). Hydroxychloroquine has also produced marked improvement in individual case reports, at a dose of 6 mg/kg/day (18). Cyclophosphamide has been used as a steroid-sparing agent, including a low-dose oral regimen of 2 mg/kg/day in children (19) and an intravenous regimen of 15 mg/kg every 2–3 weeks (extrapolated from vasculitis trials) (14). Leflunomide was reported to be safe and effective for both induction and maintenance of remission (6). 6-mercaptopurine, used as dose-adjusted maintenance therapy (60 mg/m^2^/day for 3 years), achieved steroid-free long-term remission in a series of 15 children (20).

### 3.5 Rituximab

Rituximab, a chimeric anti-CD20 monoclonal antibody, has emerged as a promising steroid-sparing option for children with refractory or steroid-dependent IPH. In a case series from the Netherlands, rituximab was described as a novel and less toxic treatment option for children with IPH (21). A recent case report further demonstrated sustained remission after the addition of rituximab in a child with steroid-dependent disease (22). A review of the literature summarized the clinical evidence supporting rituximab in immune-related diffuse alveolar hemorrhage in children (23). Dosing regimens extrapolated from rheumatoid arthritis (1 g every 2 weeks for two doses) or ANCA-associated vasculitis (375 mg/m^2^/week for 4 weeks) have been proposed (14).

### 3.6 Adjunctive and rescue therapies

In addition to corticosteroids and immunosuppressants, several adjunctive and rescue strategies have been reported. Extracorporeal membrane oxygenation (ECMO) has been successfully applied as rescue therapy for life-threatening hemorrhage (26). Arterial embolization was reported in refractory recurrent bleeding (27). Iron chelation with deferoxamine has been used to reduce pulmonary iron deposition in patients with frequent recurrences (28). In IPH associated with celiac disease (Lane–Hamilton syndrome), a gluten-free diet was beneficial (29). Mesenchymal stem cell (MSC) transplantation was reported by a Chinese group to induce complete remission in all 10 treated children (median follow-up 23 months), representing a promising exploratory therapy (30); a clinical trial of umbilical cord MSC in children with IPH has also been registered (ChiCTR1800014907). Disease recurred in all reported patients who underwent lung transplantation for end-stage IPH (14).

### 3.7 Relapse and prognosis

Despite the various therapeutic options described above, the prognosis of IPH remains guarded. Reported relapse rates were high, ranging from approximately 35% (9 of 26 children in the Kabra cohort 35) to 59.4% (a cohort of 32 children 7). The most common triggers were respiratory tract infections (37.5%) and corticosteroid tapering (18.8%), and a history of allergy was an independent risk factor for recurrence (HR 4.255, 95% CI 1.107–16.356) (7). In a multicenter Chinese cohort of 567 children with DAH, the overall mortality was 8.7%, and corticosteroid treatment for at least 3 months was associated with significantly lower mortality (5). A single-center study of 41 children reported a mortality of 27.3% among 22 followed patients, with elevated bilirubin associated with poor prognosis (31).

## 4 Discussion

This systematic review synthesized the available evidence on the treatment of pediatric idiopathic pulmonary hemosiderosis (IPH), spanning more than six decades of literature. Our findings confirm that corticosteroids remain the cornerstone of treatment, with the majority of children receiving corticosteroid monotherapy as initial therapy, whereas immunosuppressive agents were reserved for refractory, steroid-dependent, or relapsing disease.

Despite treatment, studies that reported relapse rates consistently observed substantial relapse risk, with rates ranging from approximately 35% to 60% across small single-center cohorts (7, 35). The largest multicenter cohort (*n* = 567) did not report relapse data (5), reflecting a critical evidence gap and the difficulty of collecting this outcome during long-term follow-up; current relapse estimates are therefore derived from small samples and should be interpreted with caution. This high relapse burden is broadly consistent with adult IPH, in which a comprehensive review of 84 adult patients reported recurrence in 54.5% and a short-term mortality of approximately 20% (32). The optimal duration of corticosteroid therapy remains controversial: some studies found that prolonging prednisone treatment beyond 3 years did not reduce relapse (15), whereas others recommended long-term maintenance for at least 2 years (13). Emerging agents, including rituximab, leflunomide, and liposteroid, showed promise as steroid-sparing or maintenance options, but their supporting evidence is limited to small series and case reports. At a mechanistic level, a recent hypothesis proposes that histamine, eosinophil cationic protein, and vascular endothelial growth factor mediate the alveolar capillary injury underlying recurrent hemorrhage. These mediators are released in genetically predisposed individuals upon antigen exposure. This mechanism may inform future targeted therapies (33).

Although direct head-to-head comparisons are lacking, several steroid-sparing agents have been used in children with IPH. Azathioprine is effective but carries a meaningful burden of adverse effects: in one series, three of seven treated children discontinued it because of granulocytopenia or gastrointestinal intolerance (16). Hydroxychloroquine is better tolerated but is supported by limited evidence, consisting mainly of case reports and small series (14, 16). Rituximab offers substantial steroid-sparing potential and a favorable toxicity profile, but its supporting evidence is the smallest, limited to small series and case reports (21, 22). Leflunomide appears promising for both induction and maintenance and is the only agent currently under formal evaluation in a registered phase 1/2 trial (NCT05937191, *n* = 34), with results expected in late 2026 (6). In practice, the choice among these agents has been individualized according to tolerability, adverse-effect profile, and clinical context, rather than established by comparative evidence.

From a clinical standpoint, the treatment options reported in the literature have generally been used in a stepwise manner. Corticosteroids (oral prednisone 1–2 mg/kg/day) remain the first-line option for all children with IPH (4). For children who are steroid-dependent or experience recurrent relapse, hydroxychloroquine or azathioprine may be added as steroid-sparing agents, the former being better tolerated (14, 16). For refractory or life-threatening disease, rituximab or leflunomide may be considered (6, 21, 22). In acute severe hemorrhage, intravenous methylprednisolone pulse therapy combined with high-dose intravenous immunoglobulin rapidly controlled pulmonary bleeding and achieved survival in a series of 35 children (13). These suggestions rest on low-level evidence and should be interpreted with caution until prospective studies become available.

This review has several limitations. First, the evidence was derived almost exclusively from case reports, case series, and small retrospective cohorts, with no randomized controlled trials, which limits the strength of our conclusions. Second, marked heterogeneity in treatment regimens, doses, and outcome definitions precluded a formal meta-analysis. Third, publication bias is likely, as favorable outcomes are more readily reported than negative ones. Fourth, most studies had limited follow-up, and long-term outcomes, including the risk of pulmonary fibrosis, remain uncertain. Finally, the largest multicenter cohort comprised children with DAH of heterogeneous etiology (5); among those with IPH or undetermined etiology, the boundary between true IPH and unrecognized secondary causes may be blurred, particularly in older studies for which the diagnostic workup (e.g., serologic testing for ANA/ANCA) was less comprehensive, reflecting the inherent limitations of historical data for this rare disease rather than a methodological deficiency of the present review.

## 5 Conclusion

Corticosteroids remain the first-line therapy for pediatric IPH, and immunosuppressive or biologic agents may be added for refractory, steroid-dependent, or relapsing disease. Given the absence of high-quality evidence, the lack of head-to-head comparisons between immunosuppressive agents, and the paucity of long-term outcome data (including pulmonary fibrosis and optimal treatment duration), well-designed multicenter prospective studies with standardized treatment protocols and outcome measures are urgently needed to establish evidence-based recommendations.

## Data Availability

The original contributions presented in the study are included in the article/Supplementary Material, further inquiries can be directed to the corresponding author.
