# Supplementary material for: Treatment of idiopathic pulmonary hemosiderosis in children: a systematic review
Source: Front Pediatr. 2026 Oct 8;14:1982437. doi: 10.3389/fped.2026.1982437 (PMC13687400; doi:10.3389/fped.2026.1982437)
Supplement: Supplementary file 1 [file Table1.docx]

# Supplementary Table S1 · Characteristics of the 149 Included Studies (2026-09-24; Follow-up column added 2026-09-25)

**Purpose**: Revision supplementary material (Supplementary Table S1), responding to Reviewer 2's request for "more transparent study reporting" (listing all included studies).

**Composition**: 130 English-language + 19 Chinese-language = 149 studies.

**Rule**: Only fields present in the abstract/full text are filled in; anything not reported is marked "NR" or "Pending full text" — never inferred or fabricated.

**Fields**: Author (Year) | Country | Design | N | Treatment | Key outcomes | Follow-up.

**Diagnostic criteria**: All included studies satisfied the review's inclusion criteria (Section 2.3 of the manuscript) — confirmed IPH / idiopathic DAH (hemosiderin-laden macrophages on BAL and/or lung-biopsy-confirmed DAH, with known secondary causes excluded). Studies using special diagnostic methods are noted in Key outcomes.

**Follow-up**: Reported follow-up duration/case numbers are highly inconsistent across studies; the "Follow-up" column is filled in only when the original text explicitly reports it, otherwise "NR." This heterogeneity is described in the manuscript's limitations (item 4).

**Risk of bias**: Study-by-study risk-of-bias assessment of the 16 cohort/multicenter studies is given in Supplementary Table S2; case reports/case series are not amenable to the NIH/NOS tools, and their low evidence level is uniformly described in the Discussion.

**Country**: Identified preferentially from institutional affiliation; where affiliation was unavailable, inferred from journal/known information (noted).

## Part I · English-language included studies (130)

### A. Known 65 studies (PMIDs listed in the data-extraction table; country/sample size added here)

#### A1. Cohort / multicenter studies (core data, 16 studies)

| # | Author (Year) | Country | Design | N | Treatment | Key outcomes | Follow-up |
| --- | --- | --- | --- | --- | --- | --- | --- |
| 1 | Pediatric DAH Multicenter Collaborative Group (2026) | China | Multicenter retrospective (21 hospitals) | 567 | Predominantly corticosteroids; initial corticosteroid monotherapy 78.7%, corticosteroid + immunosuppressant 9.1% | Overall mortality 8.7%; significantly lower mortality with corticosteroid ≥3 months (P<0.01) | NR |
| 2 | Ring AM (2023) | Denmark (international) | International multicenter cohort | 124 | Routine DAH management | International pediatric DAH cohort (containing IPH data) | NR |
| 3 | Zhang Y (2018) | China | Single-center retrospective cohort (West China) | 107 | Corticosteroids in 105 | Anemia 93.5%, hemoptysis 57%; misdiagnosis rate 73.8%; most survived | NR |
| 4 | Wang L (2024) | China | Single-center cohort (West China) | 32 | Corticosteroids (± pulse) | Relapse rate 59.4%; triggers infection 37.5%, corticosteroid tapering 18.8%; allergy history HR 4.255 | NR |
| 5 | Qi Y (2021) | China | Single-center 10-year review (Fudan) | 74 | Routine DAH management | Etiology and outcomes of pediatric DAH | NR |
| 6 | Kiper N (1999) | Turkey | Cohort (1979–1994 long-term follow-up) | 23 | Long-term low-dose corticosteroids | Long-term low-dose corticosteroids prolonged survival | Long-term (1979–1994) |
| 7 | Saeed MM (1999) | USA | Cohort (CHLA, Los Angeles) | 17 | Corticosteroids | Survival better than the previously reported 2.5 years | Survival follow-up (better than prior 2.5 years) |
| 8 | Chryssanthopoulos C (1983) | Greece | Cohort | 30 | — (prognostic criteria study) | One of the earliest systematic cohorts; 18 deaths | NR |
| 9 | Kabra SK (2007) | India | Cohort (AIIMS) | 26 | Corticosteroids | Clinical features and follow-up | Follow-up (17/26 relapse-free) |
| 10 | Taytard J (2013) | France | Cohort (RespiRare) | 25 | Corticosteroids, etc. | Diagnostic delay, relapse, treatment and prognosis | NR |
| 11 | de Silva C (2018) | India | Cohort (AIIMS) | 44 | — | Etiology and outcomes of pediatric pulmonary hemorrhage | NR |
| 12 | Mopeli K (2023) | South Africa | Retrospective descriptive | 15 | — | Retrospective review of pediatric DAH at a tertiary hospital | NR |
| 13 | Wei Q (2025) | China | Single-center retrospective (Guangxi) | 21 episodes | — | Respiratory infection is an important trigger of recurrent DAH bleeding | NR |
| 14 | Li S (2025) | China | Cohort (Qingdao) | 45 | — | Age at onset is an independent risk factor for somatic growth | NR |
| 15 | Yang CT (2020) | Taiwan | Cohort | 12 | Intensified corticosteroids | Intensified corticosteroid treatment had better prognosis | NR |
| 16 | Dearborn DG (2002) | USA | Cohort (Cleveland) | 30 (infants) | — | Infant acute pulmonary hemorrhage (associated with damp/moldy environment; included as background) | NR |

#### A2. Case series (15 studies)

| # | Author (Year) | Country | Design | N | Treatment | Key outcomes | Follow-up |
| --- | --- | --- | --- | --- | --- | --- | --- |
| 17 | Wang H (2023) | China | Case series (Sun Yat-sen Memorial) | 46 | Leflunomide | Safe and effective for induction and maintenance of remission in IPH | NR |
| 18 | Terheggen-Lagro SW (2022) | Netherlands | Case series (Amsterdam) | — | Rituximab | A novel, less toxic treatment option for pediatric IPH | NR |
| 19 | Gkogkou E (2020) | Belgium | Case series | 5 | — | Report of 5 infantile DAH cases | NR |
| 20 | Park JA (2015) | USA | Case series | 6 | Intrapulmonary recombinant factor VIIa | Intrapulmonary factor VIIa for pediatric DAH | NR |
| 21 | Lapena S (1985) | Spain | Case series | 6 | Prednisone 1–2 mg/kg/day | Risk factors and treatment | NR |
| 22 | Saha V (1993) | India | Case series | — | Long-term prednisolone | Long-term corticosteroid treatment of pediatric IPH | NR |
| 23 | Bagnato L (1986) | Italy | Case series | — | Immunosuppression | Long-term evaluation of immunosuppressive treatment in pediatric IPH | NR |
| 24 | Khemiri M (2008) | Tunisia | Case series | 10 | — | IPH should prompt screening for celiac disease | NR |
| 25 | Doi T (2013) | Japan | Case series (Kyushu) | — | Liposteroid | Long-term liposteroid treatment effective | NR |
| 26 | Xu LH (2017) | China | Case series (Sun Yat-sen Memorial) | — | Corticosteroids + leflunomide + mesenchymal stem cells | Combined treatment for pediatric IPH | NR |
| 27 | Pal P (2019) | India | Case series | — | Early initiation of steroid-sparing agents | Early addition of steroid-sparing agents | NR |
| 28 | Dai JJ (2017) | China | Case series (Fudan) | 4 | Corticosteroids | Four cases of pediatric DAH | NR |
| 29 | Luo XQ (2008) | China | Case series (First Affiliated, Sun Yat-sen) | — | 6-mercaptopurine | 6-MP maintenance treatment (China) | NR |
| 30 | Sethi GR (2010) | India | Case series | — | Gluten-free diet | Lane-Hamilton: benefit from gluten-free diet | NR |
| 31 | Blanco A (1994) | Spain | Case series | 4 | — | ANCA testing in IPH children (pathogenesis background) | NR |

#### A3. Case reports (23 studies)

| # | Author (Year) | Country | Design | N | Treatment | Key outcomes | Follow-up |
| --- | --- | --- | --- | --- | --- | --- | --- |
| 32 | Vaish H (2025) | India | Case report | 1 | Methylprednisolone pulse + oral maintenance | Respiratory distress improved after pulse therapy | NR |
| 33 | Fang Y (2021) | China | Case report (Shanghai) | 1 | Corticosteroids | Corticosteroid-induced myopathy (safety warning) | NR |
| 34 | Aldaithan A (2026) | Kuwait | Case report | 1 | Rituximab (steroid-sparing) | Sustained remission after add-on for steroid-dependent/refractory disease | NR |
| 35 | Cengiz DA (2025) | Turkey | Case report | 1 | Hydroxychloroquine | Hydroxychloroquine-induced neuropsychiatric adverse effects (warning) | NR |
| 36 | Matsumoto S (2019) | Japan | Case report + literature review | 1 | ECMO | ECMO support is feasible for IPH-related DAH | NR |
| 37 | Garancini N (2026) | Italy | Case report + literature review | 1 | Arterial embolization | Arterial embolization can be considered for refractory recurrent bleeding | NR |
| 38 | AlJassmi AM (2020) | UAE | Case report | 1 | Corticosteroids | Delayed diagnosis and autoimmune background | NR |
| 39 | Chan J (2023) | USA | Case report | 1 | Corticosteroids | IPH easily misdiagnosed as hemolytic anemia | NR |
| 40 | Gabhale S (2024) | India | Case report | 1 | Corticosteroids | Rare late-onset case | NR |
| 41 | AlAgeel R (2026) | Saudi Arabia | Case report + literature review | 1 | Corticosteroids | Challenging case where history misled the diagnosis | NR |
| 42 | Khaled ZA (2025) | Palestine | Case report | 1 | Corticosteroids | Case and treatment course | NR |
| 43 | Li WX (2026) | China | Case report (Lanzhou) | 1 | Prednisone | Hereditary spherocytosis coexisting with IPH; watch for coexisting hematologic disease | NR |
| 44 | Qin L (2021) | China | Case report (Children's Hospital, Zhejiang) | 1 | Corticosteroids | IPH complicated by pulmonary nocardiosis (opportunistic infection warning) | NR |
| 45 | Huang SH (2003) | Taiwan | Case report | 1 | Low-dose cyclophosphamide | Long-term low-dose cyclophosphamide safe and effective | NR |
| 46 | Sant'Anna CC (2007) | Brazil | Case report | 1 | Azathioprine | Azathioprine for pediatric IPH | NR |
| 47 | Almeida MP (2006) | Portugal | Case report | 1 | Hydroxychloroquine | Marked improvement with hydroxychloroquine | NR |
| 48 | Viera SE (1996) | Brazil | Case report | 1 | Cyclophosphamide + prednisone | Cyclophosphamide + prednisone treatment | NR |
| 49 | Sun LC (2006) | Taiwan | Case report | 1 | ECMO | ECMO rescue of a child with IPH | NR |
| 50 | Watson AM (2004) | USA | Case report (Mayo) | 1 | Corticosteroids | Legionella pneumonia during immunosuppression (infection risk) | NR |
| 51 | Ben Halima N (2003) | Tunisia | Case report | 2 | Inhaled corticosteroids | Inhaled corticosteroids for IPH | NR |
| 52 | Stalder G (1959) | Switzerland | Historical case | 1 | High-dose intermittent prednisolone | One of the earliest reports of pediatric IPH treatment | NR |
| 53 | Testa ME (2012) | Argentina | Case report (Teper group) | 1 | Gluten-free diet | Celiac-related pulmonary hemorrhage | NR |
| 54 | Li YT (2017) | China | Case report (Third Affiliated, Sun Yat-sen) | 1 | Methylprednisolone pulse | Methylprednisolone pulse rescued fatal pulmonary hemorrhage | NR |

#### A4. Reviews / surveys (11 studies)

| # | Author (Year) | Country | Design | N | Treatment | Key outcomes | Follow-up |
| --- | --- | --- | --- | --- | --- | --- | --- |
| 55 | Saha BK (2022) | USA | Scoping review | 12 (pooled) | Liposteroid | Systematic summary of existing literature on liposteroid for IPH | NR |
| 56 | Zhang X (2026) | China | Review (Guangxi) | — | Rituximab | Summarizes clinical evidence for rituximab in immune-related DAH | NR |
| 57 | Saha BK (2022) | USA | Review | — | — | Differential diagnosis workflow between IPH and rheumatic/autoimmune DAH | NR |
| 58 | Saha BK (2020) | USA | Review | — | — | State-of-the-art comprehensive IPH review | NR |
| 59 | Saha BK (2022) | USA | Review | — | — | IPH 2022 progress review | NR |
| 60 | Tryfon S (2022) | Greece | Literature review | 80 (44 children) | Corticosteroids + gluten-free diet | Lane-Hamilton syndrome; 14% mortality in acute phase | NR |
| 61 | Ioachimescu OC (2004) | USA | Classic review | — | — | "IPH revisited" authoritative review | NR |
| 62 | Godfrey S (2004) | Israel | Review | — | — | Review of pediatric pulmonary hemorrhage/hemoptysis | NR |
| 63 | Avital A (2000) | Israel | Review | — | — | Review of pediatric pulmonary hemorrhage syndromes | NR |
| 64 | Chin CI (2015) | USA | Physician survey | — | — | Marked worldwide variation in IPH management (corroborates evidence scarcity) | NR |
| 65 | Castellazzi L (2016) | Italy | Review | — | — | Early diagnosis strategies for IPH | NR |

### B. Newly added 65 studies (re-run search, 2026-09-24)

Source: re-run of the PubMed pre-2019 search (true hits 266, of which only 121 were originally screened) plus the post-2020 search, supplemented per Section 2.3 criteria (including single-case reports).

#### B1. Case series (≥3 cases, 6 studies)

| # | Author (Year) | Country | Design | N | Treatment | Key outcomes | Follow-up |
| --- | --- | --- | --- | --- | --- | --- | --- |
| 66 | Yao TC (2003) | Taiwan | Case series | 5 | Predominantly corticosteroids (variable regimens) | 5 cases over 25 years; highly variable clinical course; mean diagnostic delay 9 months | NR |
| 67 | Zhang X (2010) | China | Case series (Fudan) | 28 | Glucocorticoids (1989–2009) | 88.5% onset before age 10; classic triad 57.1%; pulmonary hemosiderin-laden macrophages 60.7% | NR |
| 68 | Kjellman B (1984) | Sweden | Case series | 10 | Prednisone ± iron | Onset 1950–1979; prednisone mostly beneficial for acute episodes, long-term effect uncertain | Long-term (onset 1950–1979) |
| 69 | Ceruti Danus E (1980) | Mexico | Case series | 10 | Prednisone + 6-mercaptopurine | Follow-up 7.4 years; 1 of 9 uncontrolled; tapering/discontinuation associated with relapse | Follow-up 7.4 years |
| 70 | Valassi-Adam H (1975) | Greece | Case series | 31 | — (immunologic study) | Selective elevation of serum IgA in IPH children | NR |
| 71 | Barrio Corrales F (1976) | Spain | Case series | 3 | Corticosteroids; 1 also immunosuppressant | 1 well without treatment at 5 years; 1 required immunosuppression; 1 well on short follow-up | Follow-up (1 well at 5 years off treatment) |

#### B2. Single / 2-case reports (45 studies)

| # | Author (Year) | Country | Design | N | Treatment | Key outcomes | Follow-up |
| --- | --- | --- | --- | --- | --- | --- | --- |
| 72 | Meral A (1997) | Turkey | Case report | 1 | High-dose methylprednisolone failed → chloroquine 250 mg/day | Remission on chloroquine, discontinued after 1 year; sustained remission since 1994 | Long-term remission (since 1994) |
| 73 | Dua T (2000) | India | Case report | 2 | Corticosteroids → chloroquine | Both improved | NR |
| 74 | Chiu JH (2001) | Taiwan | Case report | 1 | Prednisolone → add-on immunosuppressant | Relapsing hemoptysis required add-on maintenance | NR |
| 75 | Coutinho MB (2002) | Portugal | Case report | 1 | Hydroxychloroquine for 2 years | Unilateral sensorineural hearing loss (ototoxic adverse effect) | NR |
| 76 | Derbent M (2002) | Turkey | Case report | 1 | Standard corticosteroid 2 mg/kg/day ineffective → high-dose short course | High-dose short-course corticosteroid controlled 2 acute bleeds | NR |
| 77 | Kocakoc E (2003) | Turkey | Case report | 1 | Prednisolone | Symptom improvement | NR |
| 78 | Cavalieri S (1963) | Italy | Case report | 1 | Deferoxamine + corticosteroid (per title) | Pending full text (no abstract) | Pending full text |
| 79 | Rossi GA (1992) | Italy | Case report | 1 | Long-term prednisone + azathioprine (per title) | Pending full text (no abstract) | Pending full text |
| 80 | Niimi A (1992) | Japan | Case report | 2 | Corticosteroids | CT analysis in remission after acute episodes | NR |
| 81 | Ferrari GF (2000) | Brazil | Case report | 1 | High-dose corticosteroids | Good early response; re-bleeding and death at 2.5 months | Follow-up 2.5 months (death) |
| 82 | Colombo JL (1992) | USA | Case report | 1 | Corticosteroid + azathioprine failed → cyclophosphamide | Cyclophosphamide for 14 months, remission nearly 5 years | Remission nearly 5 years |
| 83 | Chen KC (2004) | Taiwan | Case report | 2 | Corticosteroids | Both doing well, regular follow-up | Regular follow-up |
| 84 | Vinodh BN (2006) | India | Case report | 2 | Oral corticosteroids | Good response | NR |
| 85 | Naithani R (2006) | India | Case report | 1 | Corticosteroid + chloroquine; massive bleed at 4 years → cyclophosphamide | Cyclophosphamide rescued fatal bleeding | NR |
| 86 | Sen T (2008) | India | Case report | 1 | Oral corticosteroids | Complete clinical + radiologic remission | NR |
| 87 | Picard E (2008) | Israel | Case report | 1 | — (serial pulmonary function measurement) | Pending full text (no abstract) | Pending full text |
| 88 | Kamienska E (2009) | Poland | Case report | 1 | Corticosteroids | Partial remission | NR |
| 89 | Bhatia S (2011) | India | Case report | 1 | Corticosteroids (anemia-predominant) | Pending full text (truncated abstract) | Pending full text |
| 90 | Poggi V (2011) | Italy | Case report | 1 | Prednisolone 2 mg/kg/day | No re-bleeding after treatment | NR |
| 91 | Ibrahem R (2011) | UK | Case report | 1 | Intermittent corticosteroids | Symptoms not fully controlled | NR |
| 92 | Flanagan F (2012) | Ireland | Case report | 1 | Intravenous cyclophosphamide | First successful case of chronic idiopathic capillaritis | NR |
| 93 | Sankararaman S (2012) | USA | Case report | 1 | Corticosteroids | Early diagnosis and treatment can prevent re-bleeding (diagnostic challenge) | NR |
| 94 | Afzal N (2012) | Pakistan | Case report | 1 | Corticosteroids | Improvement and survival despite severe anemia (first Pakistan case) | NR |
| 95 | Gutierrez S (2013) | USA | Case report | 1 | ECMO + high-dose corticosteroids | Conventional ventilation failed, ECMO successful | NR |
| 96 | Eldem İ (2015) | Turkey | Case report | 1 | Corticosteroids + iron | Clinical + laboratory improvement | NR |
| 97 | Chen CC (2016) | USA | Case report | 1 | Corticosteroids | Anemia/jaundice/developmental delay onset (outcome not detailed) | NR |
| 98 | Watanabe H (2015) | Japan | Case report | 1 | Prednisolone | Good response (Down syndrome) | NR |
| 99 | Erkoçoğlu M (2016) | Turkey | Case report | 1 | Corticosteroids (coexisting IgA deficiency) | Pending full text (treatment not detailed) | Pending full text |
| 100 | Doğruel D (2017) | Turkey | Case report | 1 | Corticosteroids | Hemolytic-anemia-like onset | NR |
| 101 | Ahmed M (2017) | India | Case report | 1 | Intravenous methylprednisolone pulse | Recovery after acute event | NR |
| 102 | Temel MT (2019) | Turkey | Case report | 1 | Corticosteroids | Rapid improvement | NR |
| 103 | de Klerk KD (2018) | Namibia | Case report | 1 | Corticosteroids ± immunosuppression | Requires long-term follow-up | Requires long-term follow-up |
| 104 | Sasaki K (2019) | Japan | Case report | 1 | — (Hajdu-Cheney syndrome) | Fatal (no abstract, per title) | Pending full text |
| 105 | Asseri AA (2019) | Saudi Arabia | Case report | 1 | Alternate-day prednisolone + azathioprine + hydroxychloroquine | Complicated by pulmonary embolism | NR |
| 106 | Michaeli J (1987) | — | Case report | 1 | Immunosuppression | Worsening in pregnancy, intrauterine fetal death at 28 weeks | NR |
| 107 | O'Donohue WJ (1974) | USA | Case report | 1 | Cyclophosphamide (per title) | Pending full text (no abstract) | Pending full text |
| 108 | Carnelli V (1979) | Italy | Case report | 1 | Cyclophosphamide (per title) | Pending full text (no abstract) | Pending full text |
| 109 | Kennedy WP (1966) | UK | Case report | 1 | Prednisone (per title) | Pending full text (no abstract) | Pending full text |
| 110 | Garatti M (1983) | Italy | Case report | 1 | Cyclophosphamide | Complete hematologic + pulmonary remission at 1 year | Follow-up 1 year (remission) |
| 111 | Tutor JD (1995) | USA | Case report | 1 | Inhaled flunisolide | Successful treatment | NR |
| 112 | Ohga S (1994) | Japan | Case report | 2 | Methylprednisolone pulse (acute) → liposteroid 0.05 mg/kg (refractory) | Liposteroid stopped refractory bleeding, hemoglobin normalized | NR |
| 113 | Yacoub M (1994) | Tunisia | Case report | 2 | Gluten-free diet ± prednisone | 1 relapsed and died at 2 months | Follow-up (1 died at 2 months) |
| 114 | Pedersen FM (1996) | Denmark | Case report | 1 | Corticosteroids + cytotoxic agent | Immunosuppressive treatment | NR |
| 115 | Yeager H (1976) | — | Case report | 2 | Azathioprine | Symptom improvement; stable imaging/pulmonary function | NR |
| 116 | Ng SC (1998) | Singapore | Case report | 1 | High-dose inhaled budesonide + low-dose oral prednisolone | Symptom control | NR |

#### B3. Reviews containing original cases (2 studies)

| # | Author (Year) | Country | Design | N | Treatment | Key outcomes | Follow-up |
| --- | --- | --- | --- | --- | --- | --- | --- |
| 117 | Aceti A (2012) | Italy | Review (with original case) | 1 | Hydroxychloroquine | Down syndrome girl, hydroxychloroquine successful (source of pediatric HCQ dose) | NR |
| 118 | Bailey P (1979) | — | Review (with original cases) | 2 | Immunosuppression | 2 cases + review; immunosuppression can prolong remission | NR |

#### B4. Group B clearly included (7 studies, pre-2019 titles without "idiopathic")

| # | Author (Year) | Country | Design | N | Treatment | Key outcomes | Follow-up |
| --- | --- | --- | --- | --- | --- | --- | --- |
| 119 | Paksu S (2012) | Turkey | Case report | 1 | Gluten-free diet + corticosteroids | Lane-Hamilton; both intestinal and pulmonary symptoms controlled | NR |
| 120 | Limme B (2014) | Belgium | Case report | 1 | Prednisone 1 mg/kg/day (0.5 at discharge) | Neonatal IPH at 3 weeks | NR |
| 121 | Potalivo A (2015) | Italy | Case report | 1 | High-dose corticosteroids | Rapid improvement | NR |
| 122 | Sakamoto R (2018) | Japan | Case report | 1 | Liposteroid + methylprednisolone | Prior cumulative corticosteroid 1062 mg/kg without remission; liposteroid rescued | NR |
| 123 | Noronha N (2018) | Portugal | Case report | 1 | Corticosteroids | Iron-deficiency-anemia onset, corticosteroid treatment | NR |
| 124 | Sawiełajc K (1994) | Poland | Case report | 1 | Corticosteroids + azathioprine | Good combined-treatment effect | NR |
| 125 | Weimer B (1998) | Germany | Case report | 1 | Corticosteroids + anti-reflux measures | 1 relapse at 7 months, then remission | Follow-up (1 relapse at 7 months, then remission) |

#### B5. Post-2020 clearly included (4 studies)

| # | Author (Year) | Country | Design | N | Treatment | Key outcomes | Follow-up |
| --- | --- | --- | --- | --- | --- | --- | --- |
| 126 | Buckley M (2020) | USA | Case report + review | 1 | Immunotherapy | Differentiation of IPH mimicking vasculitis | NR |
| 127 | Mondkar SA (2022) | India | Case report | 1 | Gluten-free diet | Lane-Hamilton, 5-year-old girl | NR |
| 128 | Uejima Y (2024) | Japan | Case report | 1 | Corticosteroids ± immunosuppression | Kabuki syndrome complicated by IPH | NR |
| 129 | Gupta L (2024) | UK | Case report | 1 | Methylprednisolone (caused bradycardia) → azathioprine | Improved after switching to azathioprine | NR |

#### B6. Embase-only (1 study, no PMID)

| # | Author (Year) | Country | Design | N | Treatment | Key outcomes | Follow-up |
| --- | --- | --- | --- | --- | --- | --- | --- |
| 130 | Rosipal Š (2005) | Slovakia | Case series | 16 | — (prognostic study) | Prognosis of childhood IPH (Slovak; pending full text) | Pending full text |

## Part II · Chinese-language included studies (19)

Citations follow the CNKI official GB/T 7714 export (verified study-by-study against PDF full text on 2026-09-24).

### A. Clinical analyses / case series (12 studies, with original patient data)

| # | Author (Year) | Institution/City | Design | N | Treatment | Key outcomes | Follow-up |
| --- | --- | --- | --- | --- | --- | --- | --- |
| 131 | Zang Yan (1995) | Beijing Children's Hospital | Retrospective clinical analysis | 245 | Full-dose corticosteroids; deferoxamine for ≥4 recurrences | Earliest large Chinese series; deaths mainly from infection and bleeding | NR |
| 132 | Pan Lingli (2005) | West China Second University Hospital | Retrospective clinical analysis | 46 | Corticosteroids in 40 (prednisone 1–2 mg/kg/day) + cyclosporine in 1 + symptomatic in 6 | Corticosteroid-treated improved; 20 relapse episodes | NR |
| 133 | Yang Kun (2006) | West China Second University Hospital | Retrospective clinical analysis | 45 | All corticosteroids (severe: hydrocortisone IV first) | Misdiagnosis rate 42.2%; 5 deaths among 30 followed (4 massive hemoptysis + 1 infection) | 30 followed (5 deaths) |
| 134 | Yin Qingning (2011) | Qinghai Provincial Women and Children's Hospital | Retrospective clinical analysis | 35 | All corticosteroids; 16 also methylprednisolone pulse + IVIG | All effective, no deaths; combination group faster bleeding control, shorter stay | NR |
| 135 | Qiu Weiling / Chen Zhimin (2013) | Children's Hospital, Zhejiang University School of Medicine | Retrospective + follow-up | 41 (22 followed) | Corticosteroids in 40 | 6 deaths among 22 followed (27.3%); higher bilirubin in the death group | 22 followed (6 deaths) |
| 136 | Jiang Man (2016) | Shenzhen Children's Hospital | Retrospective clinical analysis | 10 | Prednisone 1–2 mg/kg/day; 1 pulse + 1 inhaled maintenance | 1 death from pulmonary-hemorrhage respiratory failure; median diagnostic delay 4 months | NR |
| 137 | Zhang Huiqin (2017) | Xijing Hospital | Retrospective clinical analysis | 36 | Corticosteroids (grouped by course) | Course >3 years did not reduce relapse; misdiagnosis time/initial hemoptysis were relapse risk factors | Follow-up (course >3 years assessed) |
| 138 | Zhang Hongmei (2018) | Children's Hospital Affiliated to Zhengzhou University | Retrospective clinical analysis | 20 | Glucocorticoids (pulse in acute phase) | At 2 years: cured 4/remission 7/relapse after discontinuation 5/no change 4; misdiagnosis 18/20 | Follow-up 2 years |
| 139 | Wang Youfeng (2019) | Nanyang First People's Hospital | Retrospective clinical analysis | 7 | All corticosteroids; 1 also azathioprine | All 7 clinically cured, 1 with residual pulmonary fibrosis; misdiagnosis 3/7 | NR |
| 140 | Zeng Maojun (2019) | Third Xiangya Hospital of Central South University | Case report | 3 | Prednisolone | All 3 misdiagnosed as iron-deficiency anemia (longest 6 years); hemoglobin rose after corticosteroids | NR |
| 141 | Zhuo Yufei (2020) | Hunan Children's Hospital | Retrospective clinical analysis | 19 | Corticosteroid monotherapy 7; + azathioprine 7; switched to hydroxychloroquine 3; tacrolimus/methotrexate 1 each | Monotherapy relapse 4/7; azathioprine no relapse 5/7 but adverse effects in 3; hydroxychloroquine reduced relapse | NR |
| 142 | Wu Lang (2022) | Second Affiliated Hospital of Hainan Medical University + Hainan Provincial People's Hospital | Retrospective clinical analysis | 12 | Corticosteroids first-line in 10; anti-infection in 6; transfusion in 3 | 10 cured and discharged; all 7 followed relapsed (improved on retreatment); 5 lost to follow-up | 7 followed (all relapsed) |

### B. Reviews (7 studies, as treatment evidence/background)

| # | Author (Year) | Institution/City | Design | N | Treatment | Key outcomes | Follow-up |
| --- | --- | --- | --- | --- | --- | --- | --- |
| 143 | Qin Zhaoyuan (2005) | First Affiliated Hospital, Sun Yat-sen University | Review | — | Corticosteroids + immunosuppressants | Diagnosis and treatment; source of pediatric AZA dose 1.2–2.5 mg/(kg·day) | NR |
| 144 | Ge Wei / Sun Ruopeng (2007) | Qilu Hospital of Shandong University | Review | — | — | Research progress (cited 35 times; classic Chinese review) | NR |
| 145 | Gao Susu / Yu Wencheng (2018) | Affiliated Hospital of Qingdao University | Review | — | — | Diagnosis and treatment review; contains adult data | NR |
| 146 | Jiang Tingting (2019) | Shenzhen Children's Hospital | Review | — | — | Comprehensive pediatric IPH review (rFVIIa, TPMT, MSC, etc.) | NR |
| 147 | Sun Jialiang (2019) | First Hospital of Jilin University | Review | — | — | Treatment review: corticosteroid dosing by type, CTX/AZA/6-MP/HCQ doses | NR |
| 148 | Wang Ya / Yu Jie (2022) | Children's Hospital of Chongqing Medical University | Review | — | — | Etiology and treatment | NR |
| 149 | Wei Mengyue (2023) | Beijing Children's Hospital | Review | — | — | Diagnosis and treatment progress (BAE, MSC, ECMO, lung transplant, etc.) | NR |

## Appendix · References not counted in the 149 (listed separately for reviewer tracing)

These were identified in the re-run search but are NOT counted in the formal inclusion number per the manuscript criteria; they serve as background or were excluded. This is proactively explained in the response letter.

### Appendix 1. Old background references, 17 (1956–1982 non-English, no full text for bias assessment; listed separately, not counted)

| PMID | Year | Title (original/translated) |
| --- | --- | --- |
| 13313137 | 1956 | Cortisone treatment of idiopathic pulmonary hemosiderosis |
| 13692438 | 1960 | [Primary pulmonary hemosiderosis in children] |
| 13881747 | 1962 | Idiopathic pulmonary haemosiderosis |
| 14089198 | 1963 | [Idiopathic pulmonary hemosiderosis] |
| 14134694 | 1964 | [On the therapy of idiopathic pulmonary hemosiderosis] |
| 14174765 | 1964 | Idiopathic pulmonary hemosiderosis |
| 14337853 | 1965 | Idiopathic pulmonary haemosiderosis |
| 4453432 | 1974 | [Idiopathic pulmonary hemosiderosis] |
| 4576444 | 1973 | [Clinical and anatomical aspects of idiopathic primary pulmonary hemosiderosis] |
| 4721437 | 1973 | [Treatment of idiopathic pulmonary hemosiderosis with immunosuppressives] |
| 5045899 | 1972 | [Pulmonary hemosiderosis with a normal thoracic picture] |
| 7163540 | 1982 | [Idiopathic pulmonary hemosiderosis] |
| 724263 | 1978 | [Contribution to the treatment of idiopathic pulmonary hemosiderosis] |
| 754167 | 1978 | [Idiopathic pulmonary hemosiderosis] |
| 810846 | 1975 | [Essential pulmonary hemosiderosis as a differential diagnostic problem] |
| 20655137 | 2010 | [Idiopathic pulmonary haemosiderosis in childhood: a good response to ...] |
| 31814232 | 2019 | Early diagnosis of idiopathic pulmonary hemosiderosis using gastric lavage |

### Appendix 2. Pure reviews excluded, 6 (no original patient data)

| PMID | Year | Title |
| --- | --- | --- |
| 10070562 | 1998 | Idiopathic pulmonary haemosiderosis. Epidemiology, pathogenic aspects... |
| 16473816 | 2006 | Pulmonary haemosiderosis in infants and children |
| 26101518 | 2015 | Use of Corticosteroids in the management of Idiopathic Pulmonary Hemosiderosis |
| 4968927 | 1966 | [Nosological classification and new therapeutic trends in idiopathic pulmonary hemosiderosis] |
| 7007858 | 1980 | [Idiopathic pulmonary hemosiderosis in childhood. Recent etiopathogenesis] |
| 8638326 | 1996 | [Idiopathic pulmonary hemosiderosis] |

### Appendix 3. Equivocal, 4 (excluded by decision, 2026-09-24)

| PMID | Year | Title | Reason for exclusion |
| --- | --- | --- | --- |
| 12099336 | 2002 | Juvenile idiopathic arthritis with pulmonary hemosiderosis | Secondary DAH (JIA autoimmune disease) |
| 18551948 | 2007 | Pulmonary haemosiderosis with juvenile idiopathic arthritis | Secondary DAH (JIA) |
| 32512987 | 2020 | Idiopathic pulmonary hemosiderosis - A rare cause of chronic anemia | 2 of 3 cases adult (age 26) |
| 35307363 | 2022 | Recurrence of idiopathic pulmonary hemosiderosis in adults with childhood onset | Adult (age 20, childhood onset) |

## Extraction notes (honest annotation)

1. **English 130**: 120 have abstracts; Treatment/Key outcomes taken from PubMed abstracts verbatim; 10 without abstracts (old references + Rosipal) filled by title only, with outcomes marked "Pending full text."
2. **Chinese 19**: 13 verified against PDF full text + 6 at citation level (Qin Zhaoyuan finalized, Wang Ya per CNKI official; the other 4 reviews pending full-text refinement).
3. **Country/institution**: English identified preferentially from institutional affiliation; Chinese marked by institution/city.
4. **Follow-up column (added 2026-09-25)**: filled only when the original text explicitly reports follow-up duration/case numbers; otherwise "NR"; studies without abstracts marked "Pending full text." The inconsistency of follow-up reporting is described in the manuscript's limitations (item 4).
5. **Diagnostic criteria**: uniformly satisfy the inclusion criteria (Section 2.3); not repeated per row; special diagnostic methods noted in Key outcomes.
6. **Count**: 149 = 130 English + 19 Chinese; Appendices 1/2/3 not counted (17 background + 6 pure reviews excluded + 4 equivocal excluded).
7. **Quality assessment scope**: only the 16 cohort/multicenter studies received NIH quality scores (see Supplementary Table S2); case reports/case series are not scored study-by-study, and their low evidence level is uniformly described in the Discussion.
