# Supplementary material for: Treatment of idiopathic pulmonary hemosiderosis in children: a systematic review
Source: Front Pediatr. 2026 Oct 8;14:1982437. doi: 10.3389/fped.2026.1982437 (PMC13687400; doi:10.3389/fped.2026.1982437)
Supplement: Supplementary file 2 [file Table2.docx]

# Supplementary Table S2 · Risk-of-Bias Assessment of 16 Cohort/Multicenter Studies (2026-09-24; CD items verified)

**Purpose**: Supplementary material for revision, presenting the methodological quality of the 16 included cohort/multicenter studies (i.e., those with a defined study design amenable to formal assessment).

**Instrument**: NIH Quality Assessment Tool for Case Series Studies (9 items).

**Why not the Newcastle-Ottawa Scale (NOS)**: 15 of the 16 studies are single-arm retrospective cohorts (no independent control group). The NOS requires an exposed-vs-unexposed comparison; its "selection of controls" and "comparability" items cannot be scored for such studies. We therefore used the NIH 9-item tool, which is designed specifically for single-arm case series and cohorts.

**Verification status**: Of the 9 original `CD` (cannot determine) items, 6 have been resolved to Y or N using open-access full text or abstracts (see "Verification record"); 3 remain `CD` (2 paywalled articles with no full text, and 1 whose full text also did not report follow-up duration).

## Legend

| Symbol | Meaning |
| --- | --- |
| Y | Yes (confirmable in abstract/full text) |
| N | No (confirmable in abstract/full text) |
| CD | Cannot determine (paywalled without full text, or not reported even in full text) |
| NA | Not applicable |

## NIH 9 items

| No. | Item |
| --- | --- |
| Q1 | Was the study question/objective clearly stated? |
| Q2 | Was the study population clearly and fully described? |
| Q3 | Were subjects recruited consecutively/representatively? |
| Q4 | Were subjects clearly defined (inclusion/exclusion criteria)? |
| Q5 | Were outcome measures clearly defined, valid, reliable, and implemented consistently? |
| Q6 | Was the follow-up duration sufficiently long? |
| Q7 | Were statistical methods appropriate (between-group comparisons / multivariate adjustment)? |
| Q8 | Were results reported adequately? |
| Q9 | Was confounding/bias controlled? |

## Main table (16 studies)

| # | Study (PMID) | Design | N | Q1 | Q2 | Q3 | Q4 | Q5 | Q6 | Q7 | Q8 | Q9 | Score | Overall |
| --- | --- | --- | --- | --- | --- | --- | --- | --- | --- | --- | --- | --- | --- | --- |
| 1 | Multicenter Collaborative Group 2026 (42298851) | Multicenter retrospective (21 centers, DAH) | 567 | Y | Y | Y | Y | Y | Y | Y | Y | N | **8/9** | Good |
| 2 | Ring AM 2023 (37101741) | International multicenter (26 centers, DAH) | 124 | Y | Y | Y | Y | Y | Y | N | Y | N | **7/9** | Moderate-high |
| 3 | Zhang Y 2018 (30278795) | Single-center retrospective (West China) | 107 | Y | Y | Y | Y | Y | Y* | N | Y | N | **7/9** | Moderate-high |
| 4 | Wang L 2024 (39300433) | Single-center cohort (West China) | 32 | Y | Y | Y | Y | Y | Y | Y | Y | N | **8/9** | Good |
| 5 | Qi Y 2021 (34976758) | Single-center 10-year review (Fudan, DAH) | 74 | Y | Y | Y | Y | Y | CD | Y | Y | N | **7/9** | Moderate-high |
| 6 | Kiper N 1999 (10213256) | Cohort 1979–1994 | 23 | Y | Y | Y | Y | Y | Y | N | Y | N | **7/9** | Moderate-high |
| 7 | Saeed MM 1999 (10492278) | Cohort 1972–1998 (CHLA) | 17 | Y | Y | Y | Y | Y | Y | N | Y | N | **7/9** | Moderate-high |
| 8 | Chryssanthopoulos C 1983 (6884388) | Cohort (prognostic criteria) | 30 | Y | Y | Y | CD | Y | Y | N | Y | N | **6/9** | Moderate |
| 9 | Kabra SK 2007 (17536132) | Retrospective case series (AIIMS) | 26 | Y | Y | Y | Y | Y | Y | N | Y | N | **7/9** | Moderate-high |
| 10 | Taytard J 2013 (24125570) | National database cohort (RespiRare) | 25 | Y | Y | Y | Y | Y | Y | N | Y | N | **7/9** | Moderate-high |
| 11 | de Silva C 2018 (29948735) | Chart review (AIIMS, pulmonary hemorrhage) | 44 | Y | Y | Y | Y | Y | CD | N | Y | N | **6/9** | Moderate |
| 12 | Mopeli K 2023 (37622106) | Retrospective descriptive (South Africa, DAH) | 15 | Y | Y | Y | Y | Y | N | N | Y | N | **6/9** | Moderate |
| 13 | Wei Q 2025 (40830444) | Single-center retrospective (Guangxi, RIIEAH) | 21 episodes | Y | Y | Y | Y | Y | N | N | Y | N | **6/9** | Moderate |
| 14 | Li S 2025 (40949900) | Cohort (Qingdao) | 45 | Y | Y | Y | Y | Y | Y | Y | Y | N | **8/9** | Good |
| 15 | Yang CT 2020 (32505591) | Cohort (Taiwan, 30 years) | 12 | Y | Y | Y | Y | Y | Y | Y | Y | N | **8/9** | Good |
| 16 | Dearborn DG 2002 (12205270) | Cohort (Cleveland, infant acute pulmonary hemorrhage†) | 30 | Y | Y | Y | Y | Y | Y | N | Y | N | **7/9** | Moderate-high |

\* Zhang 2018 Q6: median follow-up 3.71 years (sufficient duration), but only 34/107 (31.8%) were successfully followed up, with substantial loss-to-follow-up bias; see "Verification record."

† Dearborn 2002 studied infant acute pulmonary hemorrhage (environment-related), not IPH, and is included as background; the other 15 are IPH or pediatric DAH cohorts containing IPH.

## Verification record (resolution of CD items)

| Study | Original CD item | Result | Basis |
| --- | --- | --- | --- |
| Ring 2023 (37101741) | Q6 follow-up duration | **Y** | Full text (PMC10123512): "median (IQR) follow-up 3.2 (1.2–7.0) years" |
| Zhang 2018 (30278795) | Q6 follow-up duration | **Y (with follow-up-rate note)** | Full text (PMC6384493): "34 successfully followed up, median 3.71 (1.08–6.23) years"; ⚠️ only 34/107 (31.8%) followed up, severe loss to follow-up |
| Taytard 2013 (24125570) | Q6 follow-up duration | **Y** | Abstract: "median follow-up 5.5 years, satisfactory respiratory outcome in 23/25" |
| Mopeli 2023 (37622106) | Q3 consecutive recruitment | **Y** | Full text (PMC10446159): "screened 2614 in a pulmonary database, 18 met DAH definition, 3 excluded, 15 included" = complete consecutive retrieval |
| Mopeli 2023 (37622106) | Q6 follow-up duration | **N** | Full text: "1 year post-treatment, 9 had normal hemoglobin; 5 still in follow-up, 7 (46.7%) lost to follow-up" — 1-year follow-up with nearly half lost, insufficient |
| Wei 2025 (40830444) | Q6 follow-up duration | **N** | Full text (PMC12366232): studied in-hospital outcomes of RIIEAH episodes (18/19 remission), no long-term follow-up design |
| Qi 2021 (34976758) | Q6 follow-up duration | **Still CD** | Full text (PMC8649607) only states "outpatient follow-up collected corticosteroid courses and relapse frequency," no follow-up duration reported |
| Chryssanthopoulos 1983 (6884388) | Q4 inclusion/exclusion criteria | **Still CD** | Paywalled (Springer, DOI 10.1007/BF00441658), no PMC full text; abstract does not describe diagnostic criteria |
| de Silva 2018 (29948735) | Q6 follow-up duration | **Still CD** | Paywalled (Springer, DOI 10.1007/s12098-018-2725-x), no PMC full text; abstract does not report follow-up duration |

If the 3 remaining `CD` items must be fully resolved, Er-Gou would need institutional access to the Springer full texts (Chryssanthopoulos 1983 is a 1983 print article whose full text is essentially unobtainable; we recommend reporting it honestly as "full text not obtainable" in the table, without adjusting the score).

## Summary conclusions

1. **No high-level-of-evidence study**: all 16 are retrospective, single-arm, without an independent control group (Q9 confounding control is N for all), i.e., low grade within observational evidence.
2. **Quality stratification**: 4 "good" (8/9: 42298851, 39300433, 40949900, 32505591, all with between-group comparisons or multivariate adjustment); 8 "moderate-high" (7/9); 4 "moderate" (6/9). None scored ≤5.
3. **Common weakness**: Q9 (confounding control) is N in all 16 — an inherent limitation of single-arm retrospective studies, and the methodological basis for the manuscript's conclusion that the evidence level is low and controls are lacking.
4. **Key figures are defensible by source quality**: relapse rate (39300433, 8/9), mortality (42298851, 8/9), and misdiagnosis rate (30278795, 7/9) all derive from studies of moderate-high or better quality.
5. **Follow-up quality is clearly stratified**: core cohorts (Ring 3.2 years, Taytard 5.5 years, Zhang 3.71 years) meet the follow-up standard; Mopeli (1 year + 46.7% lost) and Wei (no long-term follow-up) are clearly insufficient and were honestly downgraded to N.

## Items still requiring full text (final, only 3)

| Study | Pending item | Reason |
| --- | --- | --- |
| Qi 2021 (34976758) | Q6 follow-up duration | Full text also does not report follow-up duration |
| Chryssanthopoulos 1983 (6884388) | Q4 inclusion/exclusion criteria | Paywalled, no full text (1983 print article) |
| de Silva 2018 (29948735) | Q6 follow-up duration | Paywalled, no full text (Springer) |

These 3 `CD` items do not change the overall conclusion that all studies are single-arm and retrospective without controls; Chryssanthopoulos 1983 is a 1983 article and we recommend reporting it honestly as "full text not obtainable" without adjusting the score.
